# Models Contribution to the Understanding of Sarcoidosis Pathogenesis: “Are There Good Models of Sarcoidosis?”

**DOI:** 10.3390/jcm9082445

**Published:** 2020-07-31

**Authors:** Valérie Besnard, Florence Jeny

**Affiliations:** 1 UMR 1272, Hypoxie & Poumon, Université Sorbonne Paris Nord, 1 rue de Chablis, 93017 Bobigny, France; florence.jeny@aphp.fr; 2 AP-HP, Hôpital Avicenne, Service de Pneumologie, 93017 Bobigny, France

**Keywords:** sarcoidosis, models, macrophage, lung, granuloma

## Abstract

Sarcoidosis is a systemic, granulomatous, and noninfectious disease of unknown etiology. The clinical heterogeneity of the disease (targeted tissue(s), course of the disease, and therapy response) supports the idea that a multiplicity of trigger antigens may be involved. The pathogenesis of sarcoidosis is not yet completely understood, although in recent years, considerable efforts were put to develop novel experimental research models of sarcoidosis. In particular, sarcoidosis patient cells were used within in vitro 3D models to study their characteristics compared to control patients. Likewise, a series of transgenic mouse models were developed to highlight the role of particular signaling pathways in granuloma formation and persistence. The purpose of this review is to put in perspective the contributions of the most recent models in the understanding of sarcoidosis.

## 1. Introduction

Sarcoidosis is a systemic disease of unknown etiology that is characterized by the formation of immune granulomas in different organs, mainly the lungs, the lymphatic system, the skin, the eye, and the heart [1]. The diagnosis consists of the association of compatible clinical, biological, and radiological signs, the histological demonstration of a granuloma characteristic of sarcoidosis, and the elimination of other causes of granulomatosis [2]. Genetically predisposed individuals are exposed to unknown trigger(s), possibly microorganisms, inorganic particles, or other environmental factors that initiate and maintain inflammatory and immune responses. Genetic susceptibility plays an important role in the pathogenesis of sarcoidosis [3]. Recently, nucleotide-binding oligomerization domain 2 *NOD2* mutations were involved in the pathogenesis of sarcoidosis, as well as in Blau syndrome (BS) and early onset sarcoidosis (EOS) [4,5,6,7]. NOD2 is a cytosolic protein that is involved in microbial cell wall components sensing and inflammation regulation through NFκB activation [8]. In a Swedish study, Rossides et al. showed an increasing risk (3.7 fold) in first-degree relatives of patient with sarcoidosis [9]. Likewise, analysis of a twin cohort revealed an 80-fold increased risk of developing sarcoidosis compared to the general population [10]. A gene–environment interaction study identified additional genes (*FCRL1*, *IL23R*) that could distinguish patients with or without Löfgren’s syndrome [11].

The epidemiology and phenotype of sarcoidosis varies highly around the world across age, sex, ethnicity groups, environmental exposure, and socioeconomic status [12,13,14,15]. In France, it is an uncommon disease, with a prevalence of 30.2/100,000 people and an incidence of 4.9/100,000 [16]. African Americans and Northern Europeans seem to be the two most affected groups [17,18]. Sarcoidosis incidence and prevalence appear higher in women around the world with some exceptions [19,20,21,22,23].

Even if sarcoidosis is considered as a benign disease, it still represents an important issue considering that mortality is higher in patients with sarcoidosis than in the general population with an average age of death of 70.4 versus 76.2, respectively [9,24]. Mortality rates are also greater in females than males and higher in black than white, Hispanic, or Asian patients [25,26]. Epidemiological studies on sarcoidosis-related mortality and associated morbidities [27] indicated that sarcoidosis patients were high at risk, in particular women, with an average age at death of 70 ± 13 years [24,25]. In the Western population, deaths are mainly due to fibrotic pulmonary damages, pulmonary hypertension (PAH), and less frequently to cardiac, neurological, and hepatic diseases [1,28,29]. The progression of sarcoidosis toward pulmonary fibrosis is considered as an evolutionary turning point that is associated with irreversible sequelae, significant morbidity, and mortality (11.3% in 7 years) [30].

Sarcoidosis presents a highly variable and unpredictable course. It is estimated that half of patients progress to spontaneous regression within a few years of diagnosis [1]. The treatment of sarcoidosis will be decided when the patient demonstrates an impaired quality of life or when the disease becomes threatening for an organ or the patient’s life. Despite the treatment, a significant number of patients will progress to a chronic and progressive form, 10% to 20% have sequelae, and mortality is estimated at 6–7% [1]. To date, there are no reliable markers predicting the progression of the disease [1]. Identifying the molecular mechanisms involved in the disease would help to develop specific treatments for patients who escape the various lines of treatment.

Sarcoidosis is distinct from other granulomatosis by the peculiarity of the granulomas location and distribution [31]. Lymphocyte infiltration and granulomas can be found in the visceral pleura, interlobular septa and bronchovascular vicinities. A mononuclear cell alveolitis induced by an unknown antigen initiates the accumulation of phagocytes that leads to the formation of discrete structures, which are composed of a central core of epithelioid cells surrounded by activated alveolar macrophages and T lymphocytes releasing inflammatory cytokines, including interleukin (IL)-1β, IL-12, IL-18, and tumor necrosis factor (TNF)-α [32,33,34,35]. The persistence of these structures leads to the formation of non-caseous granulomas with epithelioid cells [27]. Granulomas are composed of a central follicle consisting essentially of macrophages, epithelioid cells associated with giant cells, and CD4 + lymphocytes. The central follicle is surrounded by a lymphocyte crown characterized by the presence of CD8 + lymphocytes, rare B lymphocytes, and a predominance of CD4 + lymphocytes, including Th1, Th2, Th17, and T lymphocytes regulators. As granulomas persist, a lamellar hyaline collagen sclerosis surrounds the various follicles. The origin of cell-constituting granulomas remains uncertain. In tuberculosis, once bacillus phagocytosis by airways macrophages occurred, the infected macrophage may facilitate the spread of disease by migration to distal sites in the lungs. In the parenchyma, infected macrophages recruit uninfected macrophages (intra-alveolar macrophages and monocytes-derived macrophages of systemic origin) to ultimately form a granuloma. A similar process can be hypothesized in sarcoidosis. In the microenvironment of granulomas, intra-alveolar cells are present as a normal or mildly elevated total lymphocyte population, a normal percentage of eosinophils and neutrophils, and an absence of plasma cells and foamy alveolar macrophages [36]. Intra-alveolar monocytes and macrophages are known to feature cells constituting granulomas in the lung, including macrophage-derived epithelioid cells and macrophage-fused multinucleated cells [37]. 

Research models aim to be isomorphic (replication of pathological and histopathological features), homologous (identification of the pathogenic mechanisms), and predictive (testing of the efficacy and toxicity of potential novel therapeutic strategies). In sarcoidosis, the development of research models was initiated several years ago, with various degrees of success. Mainly in vitro and in vivo models brought some responses at the isomorphic and homologous levels, but they were not predictive yet. Generating sarcoidosis models is crucial considering excess mortality in sarcoidosis and the necessity to understand molecular mechanisms involved in sarcoidosis to develop alternatives therapies to corticotherapy. Developing experimental models is challenging for multiple reasons, including the following reasons: (1) causative agent(s) of the disease are unknown; (2) it is a systemic disease with different targeted organs for each patient and highly diverse presentations; (3) the multiplicity of cellular actors and their interactions; (4) genetic variability; and (5) the course of disease resolution is unpredictable amongst patients, indicating the influence of both environmental and genetic factors. Research models were developed using putative sarcoidosis causing agents, either antigens of infectious origin or inorganic particles. Several species of mycobacteria have been implicated as the origin of the immune reaction, notably *M. tuberculosis*, *M. bovis*, *M. leprae*, and *M. avium* [38]. Exposure to inorganic particles mainly causes foreign body granulomas, such as in models of chronic *beryllium* disease [39,40,41,42] or crystalline silica [43]. Nonetheless, recent epidemiologic and experimental data support the hypothesis on the role of nanoparticles in sarcoidosis [44,45,46,47,48,49,50]. In vivo models mostly use mice that do not spontaneously develop sarcoidosis. In veterinary medicine, sarcoidosis is a rare disease. To the best of our knowledge, there are very few publications of animal sarcoidosis [51,52,53,54].

The present review is not an exhaustive retrospective of all the literature on experimental granuloma formation, but it aims to put in perspective the contributions of the most recent models in the understanding of sarcoidosis.

## 2. In Vitro Models

So far, in vitro models used peripheral blood mononuclear cells (PBMCs) i.e., a mixed population of peripheral monocytes and lymphocytes reproducing an immune milieu or isolated monocytes derived into differentiated macrophages. 

Cultures of PBMCs in the presence of pathogen-coated beads (mostly mycobacterial antigens) generated granulomas-like structures composed of a mix of CD14^+^ monocytes, CD163^+^ mature macrophages, multinucleated giant cells (CD11b^+^), and T and B lymphocytes (previously reviewed in [55]). Although these models were not sarcoidosis specific, they provided an overview of the kinetics of cell interactions in the formation and progression of granulomas. 

The multi-walled carbon nanotubes (MWCT) model was tested using non-adherent murine macrophages in 3D cultures to determine whether granulomas would arise independently of an immune context [56]. Indeed, macrophage differentiation into epithelioid cells and the formation of stable aggregates were induced in response to MWCT exposure. Macrophages presented a phenotypic heterogeneity with the co-expression of M1 and M2 markers (TNFα, inducible NO synthase (iNos), and arginase 1 (Arg1) expression).

Very few in vitro models were developed with PBMCs from patients with sarcoidosis. Taflin et al. cultured PBMCs from sarcoidosis patients with sepharose beads coated with *M. bovis* bacillus Calmette–Guérin (BCG) [57]. This study demonstrated the important role of CD25^high^ Treg lymphocytes in granuloma formation [57,58]. CD4+CD25^high^ FoxP3+ cells exhibited powerful antiproliferative activity, without completely inhibiting TNF-α production, indicating a role for Tregs in suppressing the early stages of autologous granuloma formation. However, functional differences were observed in CD4^+^CD25^high^ cells from healthy subjects compared to those of patients with sarcoidosis. While healthy subjects’ CD4^+^CD25^high^ cells accelerated granuloma formation, no changes in the time course of granuloma growth were observed with sarcoidosis patient cells, indicating a defect in the suppressive function of Tregs in sarcoidosis.

Epithelioid cells and multi-nucleated giant cells (MGC) derived from the monocyte–macrophage lineage are the main component cells intervening in the architecture of sarcoidosis granulomas. The differentiation of monocytes into MGC can be induced by various stimuli, including cytokines (IL-3, IFN-γ), growth factors, lectins, and muramyl dipeptide. Monocytes from patients with sarcoidosis stimulated by the supernatant of concanavalin A-stimulated mononuclear cells showed an enhanced potential to form MGCs compared to monocytes from healthy controls or patients with other granulomatous diseases [59]. In the same study, Mizuno et al. showed that MGC formation from macrophages, induced by M-CSF treated-monocytes, was also significantly greater in sarcoidosis patients than in other groups, suggesting that PBMCs in sarcoidosis patients have a propensity to differentiate into MGC in response to inflammatory stimuli.

Recently, new in vitro models were developed using either PBMCs exposed to purified protein derivative of *Mycobacterium tuberculosis* (PPD)-coated polystyrene beads, or microparticles generated from *Mycobacterium abscessus* (MAB) cell walls. After 7 days of culture with the PPD model, granuloma-like multicellular aggregates were formed with the presence of macrophages in the center and peripheral lymphocytes. Crouser et al. showed that macrophages from sarcoidosis patients maintained a M2 phenotype in the presence of PPD beads compared to non-coated beads, compared to control patients for whom macrophages acquired M1 characteristics [60]. Likewise, Locke et al. showed an increase in M2 polarization with the expression of CD163 and an *IL13* gene expression profile in sarcoidosis patients compared to control patients in response to PPD beads [61]. In the MAB model, granuloma formation was observed 72 h after exposure with a marked Th1 and Th17 signature and NFκB activation [62]. 

The polarization state of macrophages varies in various lung diseases and could depend on signals present within the microenvironment. While macrophage states are globally divided into classically (M1) and alternatively (M2) activated macrophages, their polarization appears as transient and reversible, depending on the milieu [63,64]. Data from the literature present different aspects of macrophage polarization in sarcoidosis. Cytokine production in sarcoidosis was initially featured by an increase in Th1 cytokines (IL-2, IL-6, IL-12, IL-18, TNFα, IFNγ), which is potentially responsible for the so-called classical (M1) macrophage activation [65,66,67]. However, studies on marker expression on macrophages gave very opposite results. The gene expression of several M1 and M2 associated markers quantified in cultured total broncho-alveolar lavage (BAL) cells and sorted alveolar macrophages from sarcoidosis patients, with or without Löfgren’s syndrome, and compared to healthy subjects did not evidence any differences in alveolar macrophage polarization in sarcoidosis [68]. By contrast, in BAL, M1 macrophages (CD40^hi^ cells) were increased in sarcoidosis when compared with other ILD, whereas M2 macrophages (CD163 expressing cells) were similar [64]. Likewise, BAL cells from sarcoidosis patients displayed an elevated expression of the transcription factor, Twist1, which is an M1-associated gene compared to healthy controls. The expression of TWIST1 was inducible by M1 activation stimuli (LPS, TNFα) but not by IL-4 [69]. On the other hand, Prokop et al. identified a dominant Th2 response (IL-4, IL-13, IL-10, DNAX activation protein of 12kDa (DAP12)) with the expression of M2 markers (CD206, CD301, SOCS-1, ARG1, IL-4R, and IL-27R) on macrophages and giant cells in muscle sarcoidosis [70]. Immunohistochemical staining for CD163 expression was significantly increased in sarcoidosis sections compared with those from tuberculosis subjects [71]. Similarly, Isohisa et al. showed an increased immunostaining for CD163 in cutaneous sarcoidosis [72]. Recently, BAL cells from sarcoidosis subjects co-cultured with MSCs showed a reduction in TNF-α (pro-inflammatory M1) and an increase in IL-10 (anti -inflammatory M2) [73]. Obviously, it is quite difficult to establish a real consensus on macrophages polarization in sarcoidosis. There are some variabilities between these studies that need to be taken in consideration: macrophage origins were not always identical (lung versus muscle versus blood), suggesting a possible tissue variability; the local environment was different (PBMCs versus BAL cells versus biopsy samples), possibly affecting cell exposure to a sarcoidosis-causing agent but also the occurrence or not of cell–cell interaction; and experimentally, the culture of macrophages in a culture plate did not recapitulate or even induce phenotype alterations in comparison to macrophages studied ex vivo in a biopsy. One more point to take into account is granuloma dynamics: the structures are not stable and evolved in time for the best (resolution) or the worst (lasting profibrotic granuloma). While biopsies give an instant picture of a pathological process initiated long before, the results of cell cultures are the product of short-term experience at the scale of the formation of a granuloma, probably reflecting the cells and processes set up during granuloma initiation. 

## 3. In Vivo Models

In vivo models were developed in mice exposed to putative sarcoidosis-causing agents, either antigens of infectious origin or inorganic particles. In addition, two murine models of transgenic mice showed a spontaneous apparition of granulomatous with sarcoidosis-like aspects, indicating the potential role for particular genes/signaling pathways in sarcoidosis. 

The two main in vivo models based on infectious agents followed different strategies: the use of mycobacterial antigens without provoking tuberculosis and the exposure to Cutibacterium acnes *(C. acnes*) (formerly named *Propionibacterium acnes*, *P. acnes*), which is a commensal strain of bacteria. Since sarcoidosis patients can be reactive to some mycobacterial proteins (ESAT-6, KatG, Ag85A, SodA, or HSP) [74] and that mycobacteria antigens were detected in sarcoidosis patient granulomas [75,76,77,78], models were generated using mycobacterial peptides to study granuloma development in mouse lungs. Peptides from *M. tuberculosis* catalase-peroxidase (mKatG) and *M. tuberculosis* superoxide dismutase A (mSodA) induced pulmonary granulomatosis in mice [79,80]. Granulomas were histologically similar to those observed in sarcoidosis with the recruitment of macrophages, CD4^+^ T lymphocytes with Th1 cytokine (IL-2 and IFNγ) production, and rare B lymphocytes. The presence of serum amyloid A (SAA), a highly inducible acute-phase reactant and amyloid precursor protein, was readily detected in CD68+ macrophages and multi-nucleated giant cells from sarcoidosis patient granulomas in various organs (lung, lymph nodes, skin, liver) and in mycobacterial KatG–bead–induced granulomas in mice [79]. SAA expression appeared to be controlled by the CD3^+^ lymphocyte in part through a toll-like receptor 2 TLR2 signaling and correlated with tissue chronic inflammation and fibrosis. Huppertz et al. showed an increased expression of activated NLRP3 (NOD-like receptor (NLR) pyrin domain-containing protein 3) inflammasome components (caspase-1 and IL-1β) in lung granuloma, and increased IL-1β release of BAL cells from sarcoid patients [81]. In a mouse granuloma model induced by Trehalose 6.6′-dimycolate from *Mycobacterium tuberculosis*, granuloma formation was decreased in *Nlrp3^-/-^* mice and increased in *miR-223^-/-^* (micro-RNA downregulating NLRP3) mice compared to wild-type mice. 

The second model used *C. acnes* based on its detection in the lung and lymph nodes of patients [82,83]. Several experimental models of *C. acnes*-induced lung granulomatosis were published, each of them with differences in their experimental protocols [84,85,86,87,88,89,90,91,92,93]. Granulomas were formed by the recruitment of macrophages and CD4^+^T and CD45/B220^+^ B lymphocytes promoted by Th1 cytokines and chemokines (TNFα, IFNγ, MCP-1, IL12p40, and IL12p70) [84,85,86,87,88,89,90,91,92,93]. It can be emphasized that the various studies using *C. acnes* to induce pulmonary granulomatosis showed great variability concerning the mode of administration (sub-cutaneous, intravenous, intra-tracheal), the presence or not of adjuvant and the type of adjuvant used (incomplete Freund adjuvant, complete Freund adjuvant), the dose of *C. acnes*, the number of challenges, and the date of sacrifice. Several types of bacterial strains used in these experiments were either commercially provided (ATCC^®^ 6919 and ATCC^®^ 11828) [85,86,87,88,89,90,91,92] or from the lymph nodes of sarcoidosis patients [93]. The use of antibiotics targeting endogenous *C. acnes* in mice before immunization decreases pulmonary granulomatosis [92]. Using the *C. acnes* model, Werner et al. showed that the deletion of *Myd88* or *Cybb* in mice increased the persistence of alive bacteria in the lung and enhanced granuloma formation, indicating that a defect of bacterial clearance through the impairment phagosome activity could participate in granuloma development [93]. As in the model using mKatG [79], the TLR2 response was implicated in granuloma formation induced by *C. acnes* [94]. Interestingly, Song et al. showed supporting data for the role of Th17 lymphocytes and IL-17A in granuloma formation [95]. *C. acnes* induction of lung granulomas was reduced in both *IL-17A* gene knockout mice and mice receiving an IL-17A neutralizing antibody. To conclude, the difficulties in efficiently reproducing the *C. acnes*-induced pulmonary granulomatosis is probably dependent on the mechanism of repeated antigen recognition and the activation of the CD4 + lymphocyte toward a TH1 phenotype. This mechanism calls upon many factors, which are difficult to control despite the standardization of the conditions of animal facilities. In this sense, this model also responds to the random, multifactorial character described in sarcoidosis. 

Based on the possible association between carbon nanoparticles and sarcoid-like pulmonary granulomas, rodent models of chronic granulomatous inflammation were generated by the administration of MWCTs. After pulmonary exposure to MWCTs, a marked inflammatory response was present and associated with the development of granulomas and/or fibrosis [49,96]. Depending on the physicochemical properties of MWCTs, nanomaterial was detected one year later as black aggregates in macrophages or in granuloma [97]. Yanamala et al. showed distinct and common inflammatory responses to various carbonaceous materials, including MWCTs [98]. Pulmonary granulomas were observed in mice up to 90 days after exposure to the recruitment of T lymphocytes, macrophages, and the formation of multi-nucleated giant cells [99]. Interestingly, the cytokine expression profile in the MWCT model showed a concomitant expression of Th1 (TNFα, IL18), Th2 (CCL7, CCL11), and other cytokines (CCL2, CCL9, CCL22) [99]. In another inorganic particle model using quantum dots (QDOT), particularly QD705, a cadmium-based nanoparticle, lung granulomas were also detected with high expression levels of TNFα, IL6, CXCL1, and CCL2 [100]. Comparison of both models showed that although these models were capable of recruiting macrophages, as well as T, B, and Treg lymphocytes, the innate immunity profile and the cytokine production were clearly different, with a more severe inflammation in the QDOT model [101]. Interestingly, Malur et al. showed additive effects on granuloma formation by using a combination of MWCT with microbial antigens (mycobacterial antigen ESAT-6, a T cell activator associated with tuberculosis and sarcoidosis) [102,103]. The double-hit strategy promoted increased granulomas, fibrosis, and inflammatory mediators, indicating that trigger multiplication could be responsible for granuloma persistence associated with pulmonary fibrosis. 

In the pathology of sarcoidosis, alterations in circulating lipid levels are observed, notably the reduction in HDL-cholesterol levels and ApoA1 concentrations [104,105,106]. Similarly, several proteins involved in lipid metabolism, including SAA, plastin 2, fatty acid binding protein 4, and annexin A2 [107], are expressed differently in patients than in controls. These alterations in lipid metabolism are possibly related to sarcoidosis pathology itself as well as other co-pathologies observed in sarcoidosis, such as the increasing risk of atherosclerosis in patients with sarcoidosis [106,108,109].

IFNγ is a key mediator of granuloma formation in sarcoidosis. Barna et al. showed that this cytokine altered the expression of the transcription factor PPARγ (*Peroxisome Proliferator-activated Receptor gamma*) that controls the transcription of numerous genes implicated in differentiation, proliferation, and apoptosis. PPARγ was reduced in BAL lysates and nuclear PPARγ content in macrophages, suggesting that PPARγ could be an important negative regulator of inflammation in macrophages in sarcoidosis [110]. To demonstrate the role of this transcription factor, Huizar et al. used the MWCT model in mice deleted for *Pparg* specifically in the myeloid compartment. The loss of PPARγ expression in macrophages promoted a more pronounced granuloma formation and an elevated pro-inflammatory cytokine expression in mice [111]. Using the double hit model (MWCT+ESAT6), Sanderford et al. showed that *Pparg* deletion in the myeloid compartment favored an increase in the Th1 cell population (Tbet, Stat4, and Ifnγ expressing cells), and no changes in the Th2 population in BAL cells [112]. Moreover, Th17 cells (RORγt, IL17A, CCR6 expressing cells) were recruited in response to an increased expression of IL-6, CCL20. Additional studies demonstrated that cholesterol transporters ABCA1 and ABCG1 expressions were also altered in macrophages from patients with sarcoidosis [113]. Interestingly, the microRNA miR-33, a cholesterol homeostasis regulator, was increased in both BAL cells from MWCNT-instilled mice and patients with sarcoidosis. One direct target of miR-33 is the early B-cell factor 1 (*EBF1*) that could bind and activate the promoter of master regulators of adipocyte, including CCAAT-enhancer-binding proteins C/EBPα and PPARγ [114,115]. However, Barna et al. were not able to show a direct link between miR-33 expression and PPARγ reduction in granuloma macrophages [113]. 

Previous studies reported an important role of the apolipoprotein ApoE in maintaining normal lung homeostasis during lung development [116] and in response to lung injuries [117,118,119] by preventing inflammation and oxidative stress caused by hypercholesterolemia. In addition, ApoE enhances host defense to mycobacterial infection by facilitating the presentation of exogenous mycobacterial lipid antigens. After mycobacterial lipid antigens bind ApoE, the complex is internalized via the low density lipoprotein receptor LDLR into antigen-presenting cells, such as dendritic cells and activated human B cells, and then presented by the major histocompatibility complex-like molecule, CD1, to activate NKT cells [120,121]. *ApoE*-deficient mice fed a high-fat diet containing cholate developed sarcoidosis-like granulomas without being exposed to any external causing agents [122]. Epithelioid granulomas were observed in the lungs as well as in extrapulmonary locations, including thymus, stomach, liver and skin. Interestingly, fibrotic lesions occurred around granulomas with time, somewhat mimicking a chronic persistent sarcoidosis. Both ApoE deficiency and cholate diet were responsible for granuloma formation, while the high-fat diet alone induced no lesions. Interestingly, ApoE is transcriptionally regulated by PPARγ through an indirect mechanism using a liver X receptorLXR binding site present in an enhancer regulating the *ApoE* gene [123]. 

Taken together, these data suggest an important role of lipid metabolism in the regulation of pulmonary inflammation in sarcoidosis. Accumulation of events such as miR-33 presence or the decreased transcriptional activity of PPARγ in sarcoid granuloma macrophages may participate in the promotion of local and systemic dyslipidemia, triggering a persistent inflammatory response.

A second model of transgenic mice spontaneously developing sarcoid-like granulomas was generated with the deletion of the inhibitor tuberous sclerosis complex 2 (Tsc2) in the myeloid lineage in mice. This model demonstrated a major role of a Tsc2 downstream target, rapamycin (mTOR) complex 1 (mTORC1) and its signaling pathway in the initiation and persistence of granulomas [124]. Mice exhibited the spontaneous formation of non-caseating granulomas in multiple organs (lung, skin, ganglion). In these mice, macrophages deficient in *Tsc2* were hypertrophic, with an increased cell proliferation and a concomitant reduced apoptotic capacity. In parallel, the involvement of mTORC1 activation was found in biopsies of sarcoidosis patients. These results were supported by additional studies showing an increased expression of mTORC1 in cutaneous sarcoidosis patients [125] and an enrichment of the TOR signaling (*DDIT4*, *MLST8*, *DDIT4L*, *MTOR*) familial cases of sarcoidosis [126]. Interestingly, in a case report of a patient who had developed a systemic *de novo* sarcoidosis after liver transplantation, the use of rapamune, an immunosuppressor that binds to the specific cytosolic protein FKPB-12 inhibiting mTOR activation, improved dramatically the patient status with both normal chest imaging and liver biopsy [127].

## 4. Predictive Models to Test Potential Therapy

So far, the multiplicity of research model tried to replicate both pathological and histopathological features of sarcoidosis. However, very few were used as a predictive tool to test the toxicity/efficacy of potential new pharmaceutical molecules or combination of existing treatments.

A major predictive model would be to test corticosteroids (CS) resistance. Failure to respond to CS therapy is a common feature of chronical inflammatory diseases, including sarcoidosis, asthma, inflammatory bowel diseases, rheumatoid arthritis, organ transplant rejection, and chemotherapy [128,129,130,131]. Mechanisms underlying CS resistance are poorly understood. The effects of CS are mediated by the CRα receptor, whereas the CRβ displays an inhibitory role [132]. Several lines of evidence suggest that CS resistance could be due to a combination of genetic variations of the CRα receptor, including alterations in its expression levels or activation status [129]. Accumulation of the repressive form CRβ in response to inflammatory cytokines, including the Macrophage migration inhibitory factor MIF, TNF-α, IL-1β IL-8, and IL-2 in association with IL-4, could contribute to reduce CS responsiveness [133,134,135,136]. Additionally, an excessive activation of intracellular signaling pathways in response to inflammatory cytokines could reduce CS sensitivity by limiting CRα binding on the glucocorticoid-responsive elements (GREs) of target genes. The formation of CR/STAT5 heterodimers reduced the access to glucocorticoid-responsive elements (GREs) by inhibiting the CR nuclear import, causing steroid insensitivity [137,138]. Recently, the role of the Janus kinase/signal transducers and activators of transcription JAK/STAT pathway was associated with the pathogenesis of sarcoidosis [139]. The use of JAK inhibitors was reported to improve significantly sarcoidosis patient status, both at the pulmonary and cutaneous level [125,140]. Interestingly, in a specific subset of T-cell acute lymphoblastic leukemia, inhibition of the IL-7 receptor/JAK/STAT signaling by JAK inhibitors treatment enhanced the efficacy of CS by modulating the expression of genes involved in apoptosis [141]. Using research models to identify existing or new molecules in combination with CS to re-sensitize sarcoidosis granuloma cells represents a great pharmaceutical challenge.

Recently, Zhang et al. evaluated the anti-inflammatory properties of α-melanocyte stimulating hormone (α-MSH) in the MAB model [62]. α-MSH is a peptide derived from the hormone proopiomelanocortin (POMC), which is capable of reducing inflammation in endotoxin-induced uveitis and in duodenal mucosa in celiac disease patients [142,143]. The binding of α-MSH to its receptor, melanocortin receptor (MCR; MC1R and MC5R are expressed by human PBMCs), can downregulate CD86 expression on monocytes and dendritic cells and promote IL-10 release [144,145,146]. The exposure of MAB induced granulomas to α-MSH decreased inflammatory cytokines (Il1β, Il-8, CCL3, CCL5, IFNγ, GM-CSF, IL-12) without altering the granuloma structure. Two downstream targets of α-MSH appeared to be MARCO, a class A scavenger receptor and the transcription factor CREB. Pathogen Associated Molecular Pattern (PAMPs) recognition and clearing is under the control of both MARCO and TLRs. A reduction of MARCO could decrease granuloma formation. The second target of α-MSH is CREB, which is a transcription factor that can be activated by multiple signaling pathways (ERK, PKA, MSK1, JAK/STAT, GSK3b, CAMKII) in response to neurotransmitters, growth factors, ion channels, and inflammatory signals. Interestingly, CREB plays a dual role in immune responses. CREB can induce the transcription of IL-2, IL-6, IL-10, TNF-α, COX2, and MIF genes through a CRE element on their promoters. By contrast, CREB can inhibit NFκB activity by competing with CBP/p300 on their common binding site on NFκB [147,148]. Unfortunately, Zhang et al. did not evaluate NFκB DNA binding activity to confirm their hypothesis. A third potential target of the anti-inflammatory effects of α-MSH could be the JAK/STAT pathway. Indeed, when α-MSH binds to its receptor, the JAK2/STAT1 pathway can be activated [149]. 

Rosiglitazone is a selective nuclear receptor agonist of PPARγ and is an antidiabetic agent of the class of thiazolidinediones. McPeek et al. used rosiglitazone to maintain high alveolar macrophage PPARγ levels in MWCNT-induced pulmonary granulomas. Mice receiving rosiglitazone had lower MWCNT-induced pulmonary granulomas in association with a reduced pro-inflammatory response, and sustained ABCG1 expression in macrophages, supporting the concept that PPARγ deficiency could play a role in sarcoidosis pathogenesis [150].

In a different model of granulomatosis induced by Schistosoma Mansoni infection, Soliman et al. used resveratrol (RSV), a polyphenolic stilbenoid that is present in several plants [151]. Resveratrol can play beneficial roles in chronic diseases related to inflammation, including diabetes, obesity, cardiovascular diseases, and cancers. Resveratrol regulates immunity by interfering with immune cell regulation, reducing oxidative stress, reducing eicosanoids production, and by activating Sirt1, promoting the inhibition of RelA acetylation, which in turn decreases NFκB-induced expression of inflammatory factors such as TNF-α, IL-1β, IL-6, metalloproteases (MMP)-1 and MMP3, and Cox-2 [152]. Resveratrol treatment of mice infected by Schistosoma Mansoni reduced oxidative stress and the expression of the receptor for advanced glycation end products (RAGE). Interestingly, RAGE is expressed within sarcoidosis granulomas and the SNP-374 T/A polymorphism was associated with the disease [153]. Serum amyloid protein A (SAA) is implicated in sarcoidosis pathology [79,154], and it can bind to RAGE to stimulate sarcoidosis BAL cells to produce pro-inflammatory cytokines [155,156]. Testing resveratrol on sarcoidosis models would be of great interest.

Finally, new perspectives of treatment should be considered in regard to the potential role of the NLRP3 inflammasome pathway in sarcoidosis. The use of MCC950, a potent and selective inhibitor of the NLRP3 inflammasome or anti-IL-1β antibody, reduced granuloma formation [81]. 

## 5. “Are There Good Models of Sarcoidosis?”

Actual in vitro and in vivo models seek to understand the pathophysiology of the complex disease that is sarcoidosis. The need for models concerns in particular patients with poor outcomes. Future models should focus on the identification and the understanding of the mechanisms influencing the switch between spontaneous sarcoidosis resolution and treatments necessity as well as resistance/insensitivity to treatments.

**Figure 1:**
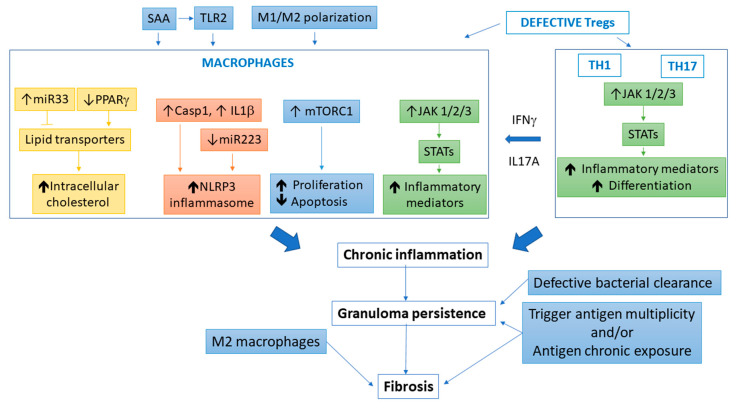
Models contribution to the understanding of sarcoidosis disease progression. In sarcoidosis, an uncontrolled pro-inflammatory phenotype takes place and appears to be amplified by the activation of both extracellular (SAA, macrophage polarization, Treg deficiency) and intracellular (TLR2, lipid metabolism, NLRP3 inflammasome, mTORC1, JAK/STAT) signaling pathways. Activation of these intracellular pathways promotes a chronic inflammatory status of macrophages. The switch “chronic inflammation/granuloma persistence” is probably due to either trigger antigens multiplicity or/and antigen chronic exposure associated with a defect in pathogen clearance. Later, the switch to the M2-like phenotype may participate to initiate or amplify the process of fibrogenesis surrounding granulomas. mTORC1, mammalian/mechanistic target of rapamycin complex 1; PPAR, peroxisome proliferator-activated receptor; SAA, serum amyloid A; TLR, toll-like receptor; Casp1, caspase 1; NLRP3, NOD-like receptor (NLR) pyrin domain-containing protein 3; JAK, Janus kinase; SAA, serum amyloid A; STAT, signal transducers and activators of transcription.

To date, research models allowed identifying and dissecting some potential interesting actors and their molecular pathways involved in antigen-driven cell aggregation/granuloma formation (Figure 1). We have a better knowledge of some biological pathways involved in granuloma formation and persistence, including macrophage polarization, implication of the mTORC signaling pathway, and the role of lipid metabolism dysregulation [60,61,62,111,112,113,124]. The use of human PBMCs from sarcoidosis patients are easily accessible, allow working on various cell populations, and demonstrate that cells have a different phenotype compared to control subjects, suggesting the expression of various genetic variants or acquired traits (epigenetics) in response to peculiar environmental pressure. Models allowed a better comprehension of the role played by the various cell subsets composing granulomas and the cell–cell interactions required to distinguish active versus inactive sarcoidosis, at the exception perhaps of the epithelioid cell population and multinucleated cells in the granuloma core, for which little research has been done so far [59]. Animal models gave more information on the complexity of the different processes, although the “mouse patient” does not always resemble to the “human patient”. Notably, transgenic mice are great tools to observe granuloma formation and evolution in response to various triggers in a more physiopathological way. Moreover, the testing of suspect antigens can be evaluated. These models permit also the use of existing treatments (rosiglitazone, rapamycin) to confirm in vivo the potential interest of these molecules in sarcoidosis treatment [124,150]. 

However, the generation of a valid and widely accepted experimental model that responds to all expectations is probably impossible. Models described so far have a number of weaknesses that would need to be addressed in futures studies:

- No spontaneous in vitro model of granuloma formation exists, and antigenic stimulation is required. Granuloma formation (in vitro and in vivo) is induced in response to agents (bacterial or inorganic origin) that are probably implicated in sarcoidosis, without certainty. In addition, certain models are more representative of foreign body granulomas (nanotube model) than sarcoidosis granulomas.
- No experimental study was done on sarcoidosis patient cells that takes into consideration the epidemiological characteristics of sarcoidosis (age, sex, ethnicity, genetics) to form distinct experimental sub-groups inside the “sarcoidosis group”. Exploration of the late stages of the disease is also missing, notably the comparison of different stages of sarcoidosis (persistent versus resolutive, active versus inactive). The generation of cell lines and/or transgenic mice expressing polymorphisms identified in sarcoidosis patients may represent great tools to understand the influence of genetic variants in the pathology.
- In vitro models are short-term studies due to the limited life span of certain cell types.
- Granuloma dynamics cannot be studied in vitro as new cell recruitment from systemic origin does not occur.
- Influence of the local environment (extracellular matrix composition, epithelial and/or mesenchymal crosstalk) remains unknown.
- Murine models of “sarcoidosis-like” granulomatosis present slightly different characteristics in terms of temporality and fibrotic evolution. It is interesting to note that exposure to sarcoidosis-causing agents generates only lung phenotypes, while genetically modified mice developed multiple sites of granulomatosis (lung, skin, liver, stomach, ganglion). In mice, local pulmonary exposure is not sufficient to spread the disease in other physiologic systems, suggesting that either the murine immune system is more efficient than humans to neutralize and eliminate pathogens, or that sarcoidosis patients are chronically exposed to one or multiple triggers, promoting granuloma development and persistence.
- Most in vivo models are restricted to a pulmonary phenotype that does not recapitulate neither the human lung disease (no PAH has been observed) nor certain extrapulmonary lesions (heart, brain, kidney, eye). Notably, no models of cardiac sarcoidosis or neurosarcoidosis exist, although they represent the two more severe organ complications after pulmonary damages.
- Fibrotic lesions were rarely observed in the various in vivo models. In sarcoidosis, fibrosis begins at the periphery of sarcoid active granulomas. Chronic inflammation is probably responsible for fibrosis extension, resulting in larger collagen deposition, and fibroblasts/myofibroblasts differentiation causing parenchymal destruction. Recently, studies have identified several MMPs in BAL fluid and the granulomas of sarcoid patients [157,158,159], possibly promoting cell migration and extracellular matrix (ECM) remodeling. MMPs are involved in pulmonary fibrosis [160]. The regulation of cytokines such as IL17 and IL22 could influence cell recruitment, granuloma formation, and lung remodeling. Other molecular actors were associated with fibrosis in sarcoidosis (>TGF-β3 (rs3917200) [161]>, IL-5 and IL-7 [162]>, S100A9 [163]>). Generating more fibrotic models could be interesting tools to identify key molecules that contribute to the pathophysiology, in order to limit their impact.
- Very few in silico models were proposed so far [164,165]. *In silico* experimentation involves the combination of biological data and mathematical and computer-based representations to granuloma models. Often, biological data are not sufficient or precise enough to establish proper mathematical models. Carrying computer-based experiments in combination with in vitro/in vivo research could facilitate not only the understanding of the disease but also the testing of new therapeutics. Humanized mice (immunodeficient mice engrafted with functional human cells and tissues) are potential preclinical animal models for the study of human diseases. The engraftment of hematopoietic stem cells from sarcoidosis patients would address not only patient uniqueness (age, sex, ethnicity, genetics…) but also experimental differences between human and murine immune systems. The engraftment of microdissected human granulomas would allow studying granuloma biodynamics in an in vivo context and potentially drug testing. Ultimately, the use of humanized mice may lead to the achievement of personalized medicine.

## 6. Conclusions

Although a great effort has been put into the development of various sarcoidosis models, there is still a lack of in vitro/in vivo models of sarcoidosis that recapitulate the human disease. A financing program was initiated by the Foundation of Sarcoidosis Research (FSR) to fund research projects developing novel experimental models to explore sarcoidosis pathogenesis and potentially the development of new therapies. Ideally, the molecular features of model granulomas would be conformed with human sarcoidosis tissue granulomas. In addition, the model should be relatively inexpensive, high-throughput, and easily manipulated experimentally. This type of sarcoidosis research model should benefit the testing of new pharmaceutical strategies intended to target a large number of patients. Nowadays, three lines of therapy are sequentially considered [166,167]. Corticosteroids are usually the first strategy of choice. When patients are intolerant or develop CS resistance, they can be switched to antimetabolics (methotrexate, azathioprine). When the first two types of treatments fail, anti-TNF-α therapy is considered. The occurrence of relapses on discontinuation or the reduction in treatment is frequent (14% to 74%) [31]. Treatment advances would benefit from innovative research models as revealed with mTOR inhibitors [127]. Alternatively, research models could be developed in order to practice a more “personalized medicine”. Testing promising treatments on models from patient PBMCs could help establish a diagnosis based on the uniqueness of each patient and to stop useless, possibly deleterious prescriptions that can be more expensive for public health care.
